# Revealing alarming changes in spatial coverage of joint hot and wet extremes across India

**DOI:** 10.1038/s41598-021-97601-z

**Published:** 2021-09-09

**Authors:** Subhasmita Dash, Rajib Maity

**Affiliations:** Department of Civil Engineering, Indian Institute of Technology Kharagpur, Kharagpur, West Bengal 721302 India

**Keywords:** Climate change, Hydrology

## Abstract

Compared to any single hydroclimatic variable, joint extremes of multiple variables impact more heavily on the society and ecosystem. In this study, we developed new joint extreme indices (JEIs) using temperature and precipitation, and investigated its spatio-temporal variation with observed records across Indian mainland. Analysis shows an alarming rate of change in the spatial extent of some of the joint extreme phenomena, tending to remain above normal. For example, above normal *hot nights and wet days* events expands at a rate of 0.61% per year considering entire Indian mainland. If the historical trend continues at the same rate, consecutive *cold and wet day* events will drop below the threshold of mean value observed in the base line period (1981–2010) everywhere in the country by the end of the twenty-first century. In contrast, the entire country will be covered by *hot nights and wet days* events only (frequency of occurrence will cross the threshold of mean value observed in the base line period). This observation is also supported by the CMIP6 climate model outputs. It is further revealed that extremes of any single variable, i.e. either precipitation or temperature (e.g., Extreme Wet Days, Consecutive Wet Days, Hot Nights, and Cold Spell Duration Index), do not manifest such an alarming spatial expansion/contraction. This indicates that the consideration of the joint indices of hydroclimatic variables is more informative for the climate change impact analysis.

## Introduction

Extreme climate events have led to a rising number of human fatalities and an exponential increase in associated damages^1–3^. Conventionally, studies on the climate extremes have mostly focused on the extremes of a single variable^4^. However, the hydroclimatic variables are interrelated, and thus, a comprehensive evaluation of the impacts owing to their extremes may fall short when analyzed individually^5,6^. Furthermore, the concurrence of extreme events in multiple climatic variables could have more serious impacts on the society and ecosystem health as compared to their occurrence in isolation^7^.

Compound climate extremes can be defined as the joint occurrence of multiple extremes eventuating simultaneously or successively for which more than one climate variables may be involved^1,6^. Some instances of the compound extremes include storm surges and wind speed^8^, hot events and droughts^9–11^, concurrence of humidity and precipitation extremes^12^, and combined risk of flooding from sea level surges and precipitation-induced high river discharge^13^. The focus of this study is based on the compound extremes of two primary hydroclimatic variables, i.e., precipitation and temperature. Individually, precipitation and temperature are associated with diverse extremes including hot extremes, cold spells, extreme precipitation, droughts and so on. However, the joint occurrence of extremes related to temperature and precipitation can impart more profound consequences than their individual occurrences^14,15^.

Owing to the climate regime shift in the 1970s, an extensive change in the characteristics of many hydroclimatic variables and associated extremes are evidenced globally^16–19^. However, at regional scales, the changes in extremes affect society more explicitly. India was placed at the 10th position among the highest climate risk countries in Asia based on the extreme climate events due to the climate change (Global Sustainable Development Report 2015). Furthermore, the large-scale circulation patterns play a crucial role towards modulating temperature and precipitation extremes worldwide including India^19,20^. Owing to the aforementioned climate shift, induced changes in the large-scale circulation patterns, such as El Niño–Southern Oscillation (ENSO), Indian Ocean Dipole (IOD) and Pacific Decadal Oscillation (PDO)^21,22^ has strongly influenced the precipitation and temperature extremes across India^23–27^. ENSO is among the most important atmospheric–oceanic mode of variability on interannual time scales, which influences the temperature and precipitation of India significantly^21,28^. El Niño (positive phase of ENSO) is dominated by precipitation deficiencies, whereas enhanced precipitation pattern is experienced during the La Niña (negative phase of ENSO). Apart from the mean climate, it is found that the ENSO and IOD substantially influence the precipitation extremes in India^29–31^. Extreme temperature variations are also found to be strongly related to ENSO^32^. For instance, the heat waves during El Niño years were found to be longer and hotter^23^. Similarly, warm (cold) phases of the PDO are associated with the decrease (increase) in the precipitation and increase (decrease) in the surface air temperature over India^22,33^.

As a possible consequence, since 1980s, India has been enduring notably warmer climate across each successive decade than the preceding decades (State of the Global Climate 2019, issued by World Meteorological Organisation, WMO) and has experienced eleven out of its fifteen warmest years since the year 2004 (https://earthobservatory.nasa.gov/images/145167/heatwave-in-india accessed in April, 2021). Furthermore, different regions of the country have been substantially affected by hot extreme events, grieved with a huge loss of lives (more than 2000) in each of the recent years, 1998, 2003, 2010, 2013 and 2015^24,34–36^. With the increasing temperature conditions, increase in the frequency and intensity of extreme precipitation events are also evidenced across India^37,38^. In the context of compound extremes, the country has experienced seven years (1951, 1972, 1979, 1987, 2009, 2014, and 2015) with concurrent hot and dry monsoonal extremes over the period of 1951–2018^39^. Furthermore, the year 2019 has witnessed the series of precipitation extremes, extreme heat spells and rarely long cold spells across different regions of India (https://www.indiatoday.in/india/story/unusual-spell-of-cold-wave-grips-north-india-here-s-why-1631803-2019-12-26 accessed in April, 2021). Thus, India being an agriculture-based country with 17.7% share of the world’s total population, the concurrence of precipitation and temperature extremes can have profound negative consequences on its socio-economic status. Objective of this study is to identify change in the characteristics of the compound extreme events associated with precipitation and temperature in the recent past (post climate regime shift) and its future (spatio-temporal) consequences across entire Indian mainland. To achieve this, first, we developed different joint extreme indices as we found that the conventional assessments are mostly based on defining the concurrence of extremes by using different thresholds^10^, which may fall short in identifying some of the apparently invisible characteristics of the compound extremes. With the newly developed indices, the impact of climate change on the spatio-temporal variation of characteristics of the compound extreme events are assessed. Secondly, spatio-temporal changes of the joint extreme characteristics before and after the climate regime shift are explored to underline the concern in the recent past. In addition, probable future changes in the joint extremes are also explored to quantify the changes under certain climate change scenarios under CMIP6.

## Results and discussion

### Proposed Joint Extreme Index (JEI) and its beneficial characteristics

**Figure 1:**
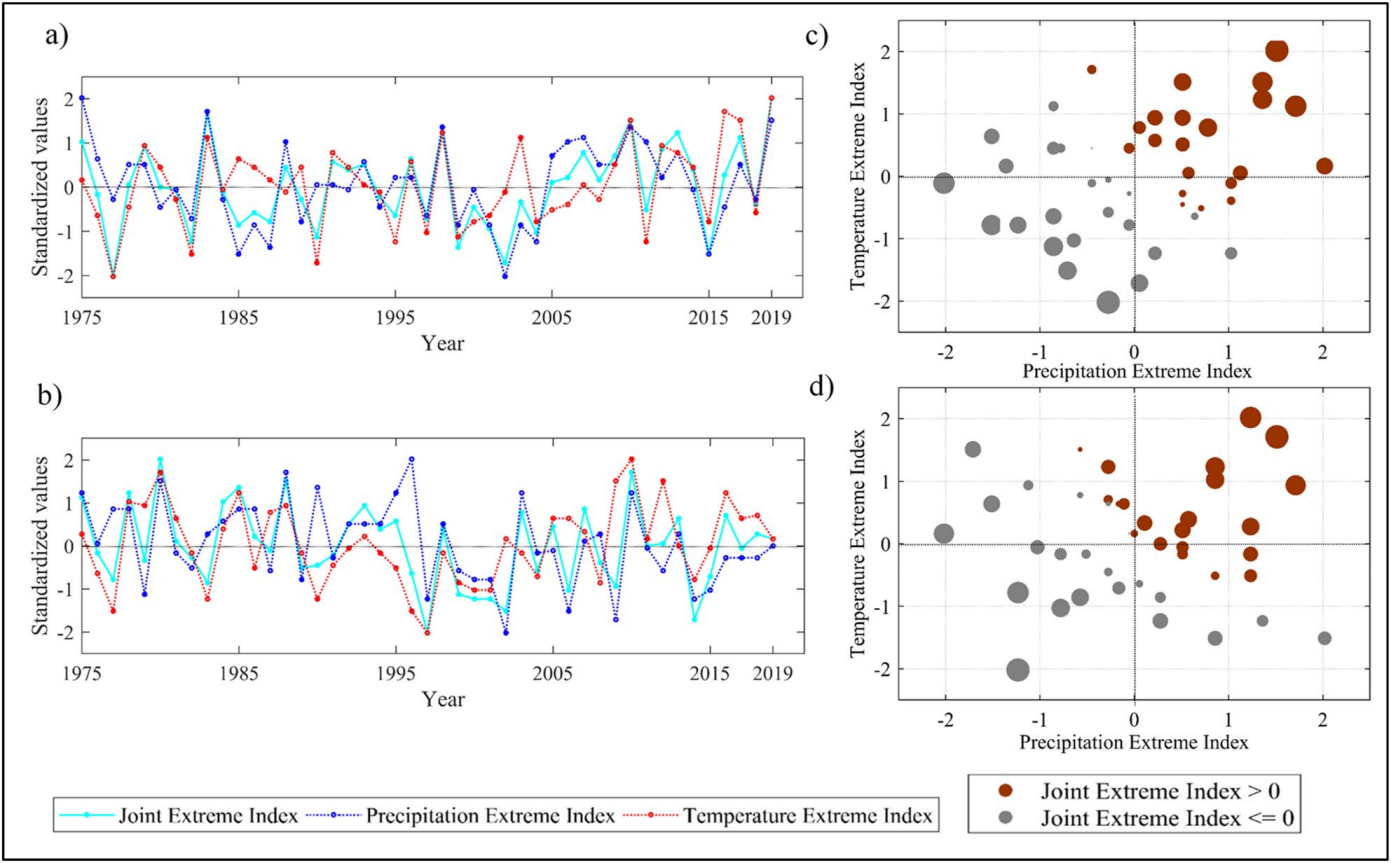
Relationship between Joint Extreme Index (JEI) and its constituting indices, i.e. Precipitation Extreme Index (PEI) and Temperature Extreme Index (TEI). (**a**) Time series of PEI (here, EWD), TEI (here, TX90) and their corresponding JEI (here, HWD) over the period 1975–2019 for South Peninsular India (SPI), (**b**) same as (**a**) but for North West India (NWI), (**c**) scatter plot of JEI on the phase plane of its constituting precipitation and temperature extreme indices. Size and colour of the circle markers indicate magnitude and phase of JEI, respectively for South Peninsular India (SPI), and (**d**) same as (**c**) but for North West India (NWI).

The results are based on the six newly proposed Joint Extreme Indices (JEIs) that characterize the joint occurrence of extreme precipitation and temperature. The developed JEIs are the standardized indicators derived using the joint distribution between precipitation and temperature based extreme indices obtained through copulas^39,41^. The six JEIs are namely Hot and Wet Days (HWD), Hot Nights and Wet Days (HNWD), Warm and Dry Spell (WDS), Warm and Wet Spell (WWS), Cold and Dry Spell (CDS), and Cold and Wet Spell (CWS). Details of their combinations and constituting indices, i.e., Temperature Extreme Indices (TEIs) and Precipitation Extreme Indices (PEIs) are shown in Table 1. Initially, we considered two regions for illustrating the properties of the JEIs in comparison with their constituting indices. These are South Peninsular India (SPI) and North West India (NWI) (shown in Figure S1) out of four homogeneous rainfall zones as defined by the India Meteorological Department (IMD) based on the coherent precipitation at regional scales^42^. The time series of HWD is shown in Fig. 1 for the two regions along with its respective TEI and PEI for discussion. The plots of other JEIs are provided in the supplementary document (Figures S2–S6) for the same regions. The joint index HWD is based on temperature extreme index, TX90 (Hot Days) and precipitation extreme index, EWD (Extreme Wet Days) (Table 1). For both the regions, in general, the JEI magnitude is noticed to be high (low) when the PEI and TEI magnitudes are high (low), which reflects the underlying attributes of the JEI in combining the status of both precipitation and temperature extremes. For instance, very high concurrent magnitudes of hot and wet days in the year 2019 in SPI (Fig. 1a) and in the year 2010 in NWI (Fig. 1b) are noticed. The regions are also noticed to have higher values of PEI and TEI during the respective years. Similarly, low JEI values are obtained for the years with low values of PEI and TEI, e.g. in the year 2015 in SPI (Fig. 1a). However, values of the JEI may or may not be high, indicating joint extreme events, when either PEI or TEI is high, depending on the combined conditions of precipitation and temperature extremes. It is true for many years. One of such instances is noticed in the year 2017 (Fig. 1a), when relatively high TEI and low PEI are noticed, however, their joint extreme is noticed to be high. In contrast, in the year 1996 (Fig. 1b), low magnitude of JEI is noticed, when TEI is very low and PEI is high. This property of the JEI indicates that the combined conditions may lead to considerable impacts, even though the contributing variables are not at extreme extent.

**Table 1:** List of joint extreme indices and corresponding precipitation and temperature extreme indices.

Joint Extreme Index (JEI)	Temperature Extreme Index (TEI)*	Precipitation Extreme Index (PEI)^#^
HWD (hot and wet days)	TX90 (Hot Days)	EWD (Extreme Wet Days)
HNWD (hot nights and wet days)	TN90 (Hot Nights)	
WDS (warm and dry spell)	WSDI (Warm Spell Duration Index)	CDD (Consecutive Dry days)
WWS (warm and wet spell)		CWD (consecutive wet days)
CDS (cold and dry spell)	CSDI (Cold Spell Duration Index)	CDD (consecutive dry days)
CWS (cold and wet spell)		CWD (consecutive wet days)

*Details in Table S2. ^#^Details in Table S3.

To further look in to the characteristics of the JEI, scatter diagram of the JEI (here HWD) on the phase plane of its constituting extreme indices (i.e., EWD as PEI and TX90 as TEI) are shown in the adjoining panels in Fig. 1 (Fig. 1c,d) for the same regions. In these plots, the marker colors differentiate between the positive and negative phases, and the marker sizes indicate the magnitude of the JEI. As obvious, four quadrants represent four different combinations of extreme precipitation and temperature conditions i.e. high PEI-high TEI, low PEI-high TEI, low PEI-low TEI and high PEI-low TEI in the 1st to 4th quadrants, respectively. The four quadrants prescribe different types of risk associated with the joint extreme events and can be described in terms of bivariate hazard scenarios^43^. A hazard scenario is defined as the set of occurrences of weather or climate events, which contribute to environmental or societal risk^44,45^. The two common scenarios employed in hydrological applications are OR and AND^46,47^. The OR scenario specifies the joint event to be extreme if either of the two constituting events (TEI and PEI) exceeds a given threshold (2nd and 4th quadrants in our case). On the other hand, both events need to exceed the threshold to fulfill the AND condition (1st and 3rd quadrants in our case). It is noticed from the scatter plots that the extreme values of the JEI belong to higher magnitudes of both the PEI and TEI in SPI and NWI (AND case). However, the reverse is not always true. Rather high values of the JEI are also obtained if it is away from a line passing through the origin and making an angle of 135° with positive x-axis (positive PEI axis). Thus, it is noticed that high JEIs are also obtained in some cases when one of the PEI or TEI is high and the other one is close to zero (OR case).

Further details regarding the developed JEIs are presented in the methodology section later. Overall, it can be recognized that the JEI provides intuitive information regarding the compound extremes and it can be a useful measure in assessing the joint extreme events associated with different precipitation and temperature extreme characteristics.

### Spatio-temporal pattern of JEI across entire India

**Figure 2:**
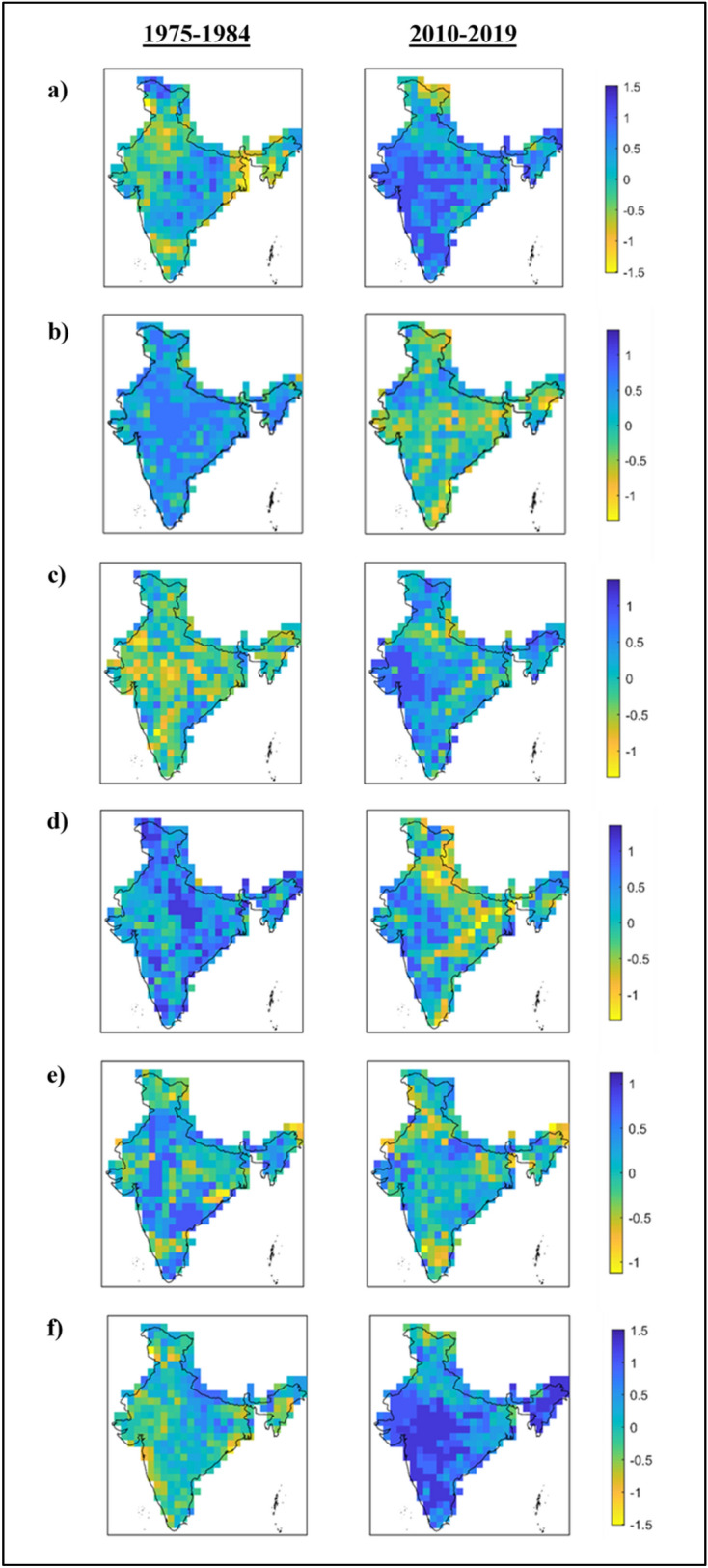
Spatial distribution of Joint Extreme Indices averaged over two decadal time periods i.e. 1975–1984 and 2010–2019 across India: (**a**) HNWD, (**b**) CWS, (**c**) WDS, (**d**) WWS, (**e**) CDS, (**f**) HWD. The figure was produced using MATLAB software (version R2021a, URL: https://in.mathworks.com).

Next, we evaluate the spatio-temporal distribution pattern of the JEI across entire Indian mainland using gridded data. Initially, we pick out two decadal periods, i.e., 1975–1984 and 2010–2019—one from the beginning of climate shift and the other from the most recent past in order to illustrate the temporal change in spatial patterns of all six JEIs. Figure 2 shows the spatial distribution of decadal average magnitude of the six JEIs across the country during the two aforementioned decadal periods. Visible changes in the spatial patterns are captured. For instance, the JEI expressing hot nights and wet days (Fig. 2a) is noticed with low magnitudes across many parts of India, especially the eastern and western regions during the past decade, which turned into exceptionally high value regions recently. Similar changes can be observed in other JEIs as well. The extent of area with the JEIs has changed notably during 2010–2019 with respect to 1975–1984. Furthermore, it is noticed that the areal extent with high magnitude of JEIs involving hot (cold) extremes has increased (decreased) across the country in 2010–2019 with respect to 1975–1984. Is it exceptional in nature and does it raise some warning inferences? To answer this, we further proceed to the quantification of spatial extent being covered by the joint extremes.

### Temporal evolution of spatial extent exhibiting Joint extreme events

**Figure 3:**
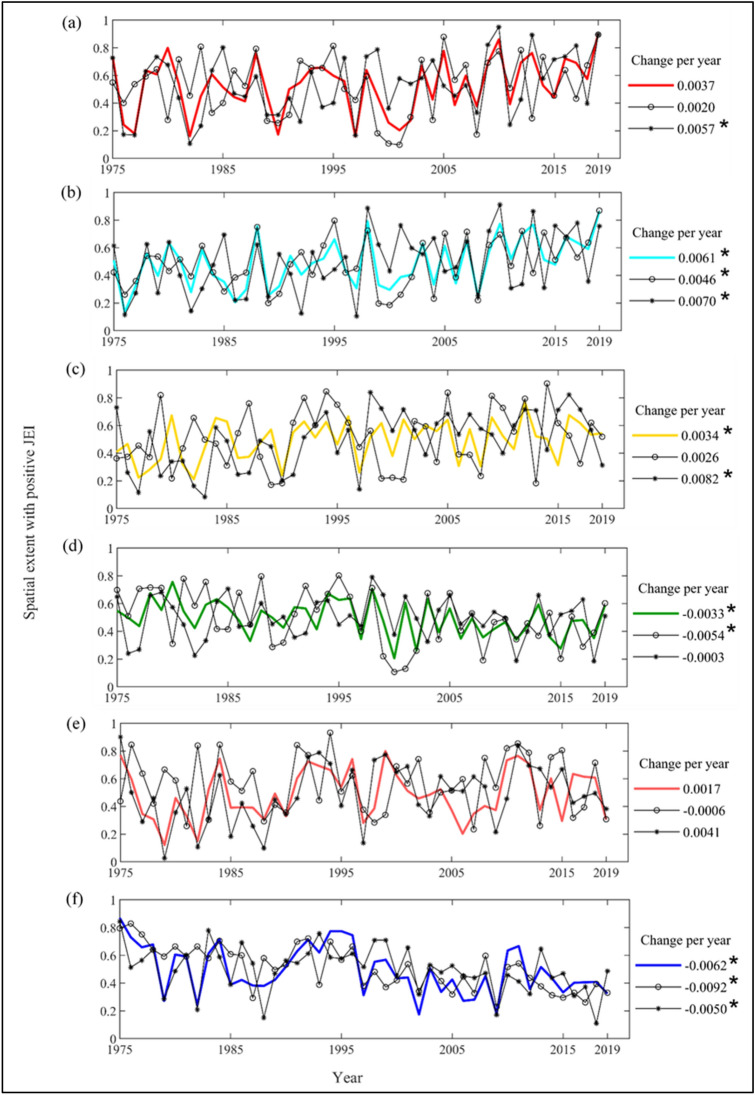
Year-wise variation of areal coverage (in proportion of entire Indian mainland) exhibiting positive Joint Extreme Indices (JEIs) during the post-climate regime shift period, i.e. 1975–2019 for three cases considering entire year (shown in solid shaded lines), monsoon season (shown in black lines with circle markers) and non-monsoon season (shown in black lines with star markers): (**a**) HWD, (**b**) HNWD, (**c**) WDS, (**d**) WWS, (**e**) CDS and (**f**) CWS. Rate of changes are shown in the legend of the respective JEI and the significant rate of change are noted with star markers as per Mann–Kendall trend test at 5% significance.

We analyzed whether the spatial extent/coverage of the joint extremes is changing over time. The gradual variation of spatial extent/coverage of different JEIs above a threshold is evaluated during the period 1975–2019 (Fig. 3). We express the spatial extent as the proportion of total area of the Indian mainland, exhibiting positive values of the JEIs. The spatial extents are estimated for three distinct cases—(1) using data from entire year, (2) using data from monsoon (June through September) season only and (3) using data from non-monsoon (October through May) season only. The temporal changes in the spatial extent are assessed using the Mann–Kendall trend analysis and significance of the changes are ascertained at 5% significance level. Notable annual as well as season-wise variation in the spatial extent are discerned corresponding to the concurrence of different precipitation and temperature extremes over the study period, i.e. post climate regime shift. Increase in the spatial extent, affected by extreme hot and wet conditions (HWD in Fig. 3a) is noticed over the years for all three cases, i.e., entire year, monsoon and non-monsoon seasons. Most rapid increase is noticed during the non-monsoon season at a rate of 0.57% per year wrt total area. Furthermore, the recent decades since 2000s have witnessed relatively higher rate of increase in the areas with positive HWD.

Spatial extent of hot nights and extreme wet days (HNWD in Fig. 3b) is also noticed to increase for the annual as well as monsoon and non-monsoon seasons. Like HWD, the non-monsoon season has exhibited more pronounced increase in the areas with positive HNWD. Other JEIs shown in Fig. 3, i.e., Fig. 3c–f, denote the joint extreme spells of temperature and precipitation, namely warm and dry spell (WDS), warm and wet spell (WWS), cold and dry spell (CDS), and cold and wet spell (CWS). The monsoon season has witnessed significant shrink in the areas with positive CWS and WWS over the years. Furthermore, out of the six JEIs, the most prominent change in spatial extent is noticed for CWS during the monsoon season i.e., at a rate of − 0.92% per year wrt total area. During the non-monsoon season, the area with CWS has also decreased significantly, whereas an insignificant decrease is noticed for the areas with WWS. In contrast, the spatial extent exhibiting warm and dry spells has significantly increased across the country and this trend is most prominent during the non-monsoon season. Opposite nature of change for the areas with warm and dry spell (increasing), and cold and dry spell (decreasing) is noticed during the monsoon season.

Overall, unprecedented changes in the spatial extent with concurrence of different precipitation and temperature extremes are observed during the recent decades, which can pose augmented impacts on the socio-economic conditions of the country. For instance, agro-economy of the country is pre-dominantly dependent on the favourable precipitation and temperature conditions during the monsoon season. However, the increase of spatial extent exhibiting warm and dry spells in lieu of the decrease of areas exhibiting cold and wet spells during the monsoon season is likely to impart substantial adverse effects on the agricultural activities across Indian mainland.

### Comparison between observed trend before and after climate regime shift in 1970s

**Figure 4:**
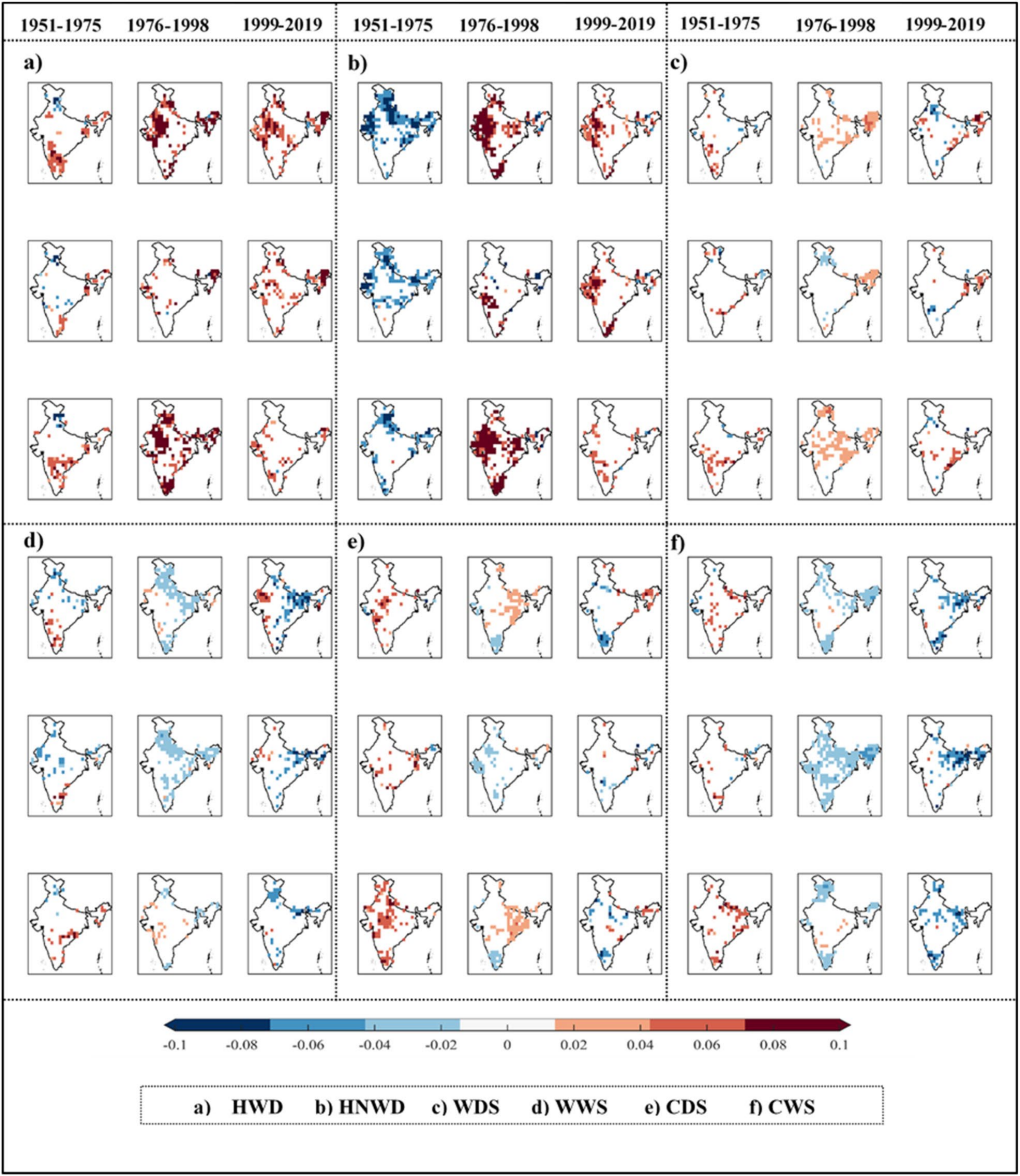
Spatial patterns of significant trends (M–K test at 5% significance) in six Joint Extreme Indices (JEIs) across Indian mainland during pre-climate regime shift period (1951–1975) and two consecutive post-climate regime shift periods consisting of early (1976–1998) and late (1999–2019) post-shift years. Each sub-plot, i.e., (**a**–**f**) (a particular JEI) shows three set (pre- and post-climate shift periods) results with the data from (top to bottom) entire year, monsoon and non-monsoon period. The figure was produced using MATLAB software (version R2021a, URL: https://in.mathworks.com).

In this section, the difference in characteristics of the JEIs before and after the climate regime shift are explored. Towards this, a pre-climate regime shift period, 1951–1975, is considered and contrasted against the post-climate regime shift periods, i.e. 1976–1998 and 1999–2019 (two separate consecutive periods consisting of early and late post-shift years, respectively). A comparison between spatial patterns with significant trends (Mann–Kendall trend test at 5% significance level) in the JEI magnitudes in these three epochs is shown in Fig. 4 considering three cases, i.e. entire year, monsoon and non-monsoon seasons. A significant change, almost diametrically opposite in some cases, is noticed. For instance, distinctly visible difference in the trend pattern of hot nights and wet days (i.e., HNWD) is noticed between the prior and later time periods with mostly decreasing trends during pre- and increasing trends during post-climate regime shift period. More areas with increasing trends are noticed during post-climate regime shift period as compared to that exhibiting decreasing trend during pre-climate regime shift period.

Hot and wet days (HWD) mostly possess an increasing trend throughout both pre- and post-climate regime shift. However, the areal extent with increasing HWD has increased significantly during non-monsoon period in the post-climate regime shift. In comparison to the areal extent exhibiting increasing hot and wet days, more areas are noticed with increasing hot night with wet day events. This indicates that the positive shift in climate warming may have caused night temperature to rise across more areas than day time temperature.

Other JEIs, such as WDS, WWS, CDS and CWS, exhibit contrasting features through their increasing or decreasing trend along with their spatial extents in the pre- and post-1976 periods. In brief, the JEIs pertaining to hot and wet conditions (HWD and HNWD) are increasing substantially in magnitude as well as areal extent across the country in the post-climate regime shift period. On the other hand, JEIs involving dry spells (WDS and CDS) are increasing mostly during non-monsoon season, whereas wet spell related indices (WWS and CWS) are found to be decreasing during the monsoon season, with substantial increase in the areal extent post the climate regime shift. Furthermore, the nature of trend noticed in the JEIs during the post-climate regime shift period was found to be consistent for the early and late post-shift periods, i.e. 1976–1998 and 1999–2019, for all the cases across the country.

### Observed trend in JEIs and future possibilities

**Figure 5:**
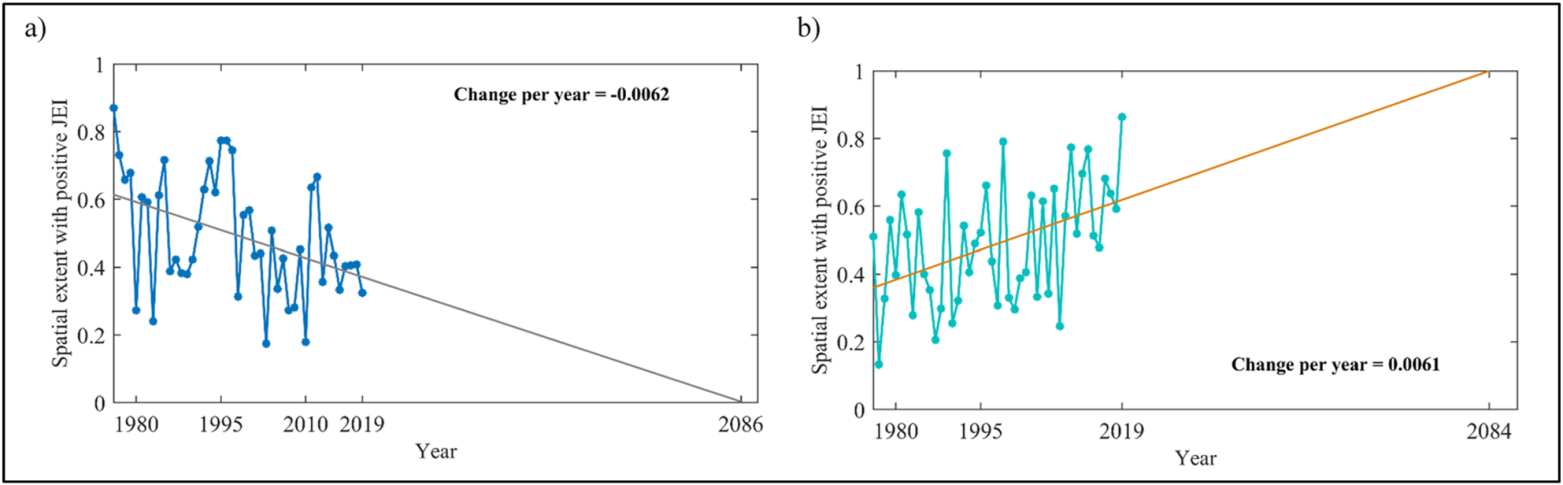
Future projection of observed significant change (during 1975–2019) in spatial extent (in proportion of entire Indian mainland) exhibiting positive Joint Extreme Indices (JEIs): (**a**) Cold and Wet Spell (CWS) and (**b**) Hot Nights and Wet Days (HNWD).

Noting the significant rate of increase in the spatial coverage by some of the joint extreme events across the country during the post-climate regime shift, we explore the future implications of the observed trend in the recent past, if it continues at the same rate. Figure 5 (and Figures S7–S9) shows the projected spatial extent exhibiting CWS and HNWD (with positive magnitudes) in the future, if the same trend prevails as observed during 1975–2019. It is noticed that the area exhibiting cold and wet spells (CWS), with the existing rate of decrease at − 0.62% per year wrt total area of Indian mainland (Fig. 3), will vanish around 2080s (2086 as shown in Fig. 5a). In other words, the joint extremes related to consecutive cold and wet day events will not be experienced anywhere in the country by around 2080s, the way it is now. It will drop below-normal, i.e., below the threshold of mean value observed in the base line period, 1981–2010, everywhere in the country by the end of the twenty-first century. On the other hand, with the prevailing increasing rate, the entire country will experience above-normal extreme hot and wet events in 2080s (2084 as shown in Fig. 5b).

Trends in the spatial coverage of the other JEIs with positive magnitudes, such as expansion of areas exhibiting warm and dry spell (WDS) and shrinkage of areas exhibiting warm and wet spell (WWS) will be significant by the end of the twenty-first century (Figure S7). Exceptionally decreasing spatial extent during the monsoon season will lead to reduced concurrence of extreme cold and wet spells below-normal in the country towards the mid of this century (Figure S8a). Similar observation is drawn for the non-monsoon season also towards the beginning of next century (Figure S9a). Furthermore, the extreme hot nights and wet days will be endured across the entire country with its above-normal occurrences by the end of this century and, in contrast, joint occurrence of the warm and wet spell will end around 2080s (Figures S8b,c) during the monsoon season. Observed changes in the spatial extent during the non-monsoon season will lead the entire country to experience above-normal occurrences of hot and wet daily extremes, and warm and dry spells around 2070s (Figure S9b–d).

### Validation of future possibilities through CMIP6 model projections

**Figure 6:**
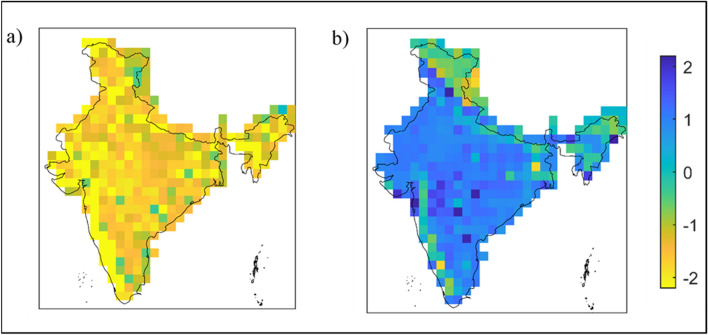
Spatial distribution of (**a**) Cold and Wet Spell (CWS) and (**b**) Hot Nights and Wet Days (HNWD) across India during the future period i.e., 2075–2084. The figure was produced using MATLAB software (version R2021a, URL: https://in.mathworks.com).

How reliable is to extend the observed historical trend to the future as the climate system is highly complex and non-linear, forced by many anthropogenic and natural forcing scenarios. Thus, validation of the future projection of joint extremes and its spatial extents is carried out using the outputs from eight General Circulation Models (GCMs), participation in the Climate Model Intercomparison Project phase 6 (CMIP6). Future projections of daily precipitation and surface temperature (minimum and maximum) are used under the worst climate change scenario (SSP5-8.5). Further details regarding the climate model simulations are discussed in the data and methodology section later. The six JEIs are estimated for the future period 2075–2084, using the model ensemble mean from the aforementioned eight GCMs. Figure 6 (and Figures S10–S12) shows the spatial distribution of magnitude of JEIs averaged over the time period 2075–2084. Considering the entire year data, the concurrence of cold and wet spell (Fig. 6a) is noticed to be below-normal almost all over the country. In contrast, above-normal occurrences of (positive magnitude) of hot nights and wet days (Fig. 6b) is noticed across all over the country apart from the Western Ghats and some parts of the Himalayan region. Thus, CMIP6 simulations support the past trend based outcomes (Fig. 5). Agreements are noticed for other JEIs as well considering entire year, monsoon and non-monsoon season (Figures S10–S12). For instance, the warm and dry spells (Figure S12b) and hot and wet days (Figure S12d) during the non-monsoon season are noticed with high magnitudes across most of the country. However, the results corresponding to the warm and wet spells are showing a contradiction to the previous findings (Figures S7a, S8b), i.e. the warm and wet spells are noticed to be above-normal (Figures S10a, S11b) across the country.

### Reason behind observed changes

**Figure 7:**
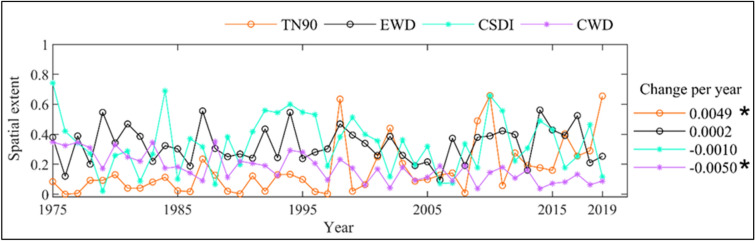
Year-wise variation of areal coverage (in proportion of entire Indian mainland) exhibiting positive Precipitation and Temperature Extreme Indices (PEIs and TEIs): TN90 (Hot Nights), EWD (Extreme Wet Days), CSDI (Cold Spell Duration Index) and CWD (Consecutive Wet Days), during the post-climate regime shift period, i.e. 1975–2019. Rate of changes are shown in the legend and the significant changes (as per M–K test at 5% significance) are noted with star markers.

Before looking into the reason behind the observed change in the joint extreme events, we first analysed the trend in the individual contributing extreme indices i.e., different PEIs and TEIs, such as TX90, EWD, CSDI and CWD (Fig. 7, Figure S13). It is noticed that the changes in some of the individual indices are of similar nature. However, the change is not as prominent as in case of their joint indices. For instance, areas exhibiting above-normal consecutive wet days (CWD) and cold spell duration index (CSDI) have been decreased during 1975–2019, which has resulted in a significant decrease of the areas exhibiting cold and wet spells in the country. Furthermore, for the JEIs having constituting indices with contrasting nature of trend, the resultant nature depends on the index exhibiting higher relative change. However, an assessment of the spatial extent of change considering all the PEIs and TEIs (Figure S13) for annual as well as monsoon and non-monsoon seasons reveals that the changes noticed in the constituting indices are not as exceptional as observed in case of their respective joint extremes for most of the cases. This observation affirms that the assessment based on the single variable may not always reveal the impacts associated with the extremes owing to multiple hydroclimatic variables that are notably associated to each other.

Remarkable changes in the joint extremes of precipitation and temperature are identified during the post climate regime shift period. These changes are perhaps the result of several mechanisms working together involving different hydroclimatic processes. Positive feedback mechanism between temperature rise (during the recent decades)^48^ and enhanced water holding capacity could be one of the possible reasons behind the changes. This conception can be justified through Clausius–Clapeyron (C–C) scaling relationship, which dictates a 7% increase in atmospheric water holding capacity for every 1 °C temperature increase^49^. Therefore, the shift in the climate towards warmer temperature in the recent past^24^, is expected to intensify the hydroclimatic extremes owing to increased moisture availability in the atmosphere. As a result, a warmer climate is likely to experience hot extremes and intense precipitation events more frequently. However, an increase in the heavy precipitation usually occur at the expense of light and moderate precipitation^50^, which leads to longer periods of consecutive dry days and shortened wet spells^51^. The land–atmosphere interaction also plays a key role towards the alterations in the precipitation and temperature extremes. For instance, warmer temperature induces higher evaporation rates, potentially drying out the soil^52^. This can amplify the surface temperature (due to formation of more sensible heat^6^) and the evaporative demand, further drying out the soil. As a consequence, this may lead to reduced precipitation owing to less evapotranspiration.

The above said mechanisms could justify the noticed changes to some extent. Furthermore, other factors, such as rapid urbanization, changing characteristics of large scale circulation patterns, such as ENSO, IOD, PDO etc. and alterations in regional hydroclimate regimes owing to the climate shift may also contribute towards the changes observed in the joint extremes. However, comprehensive exploration of the physical mechanisms responsible for these changes is beyond the scope of this study. In general, the results indicate growing risks associated with the joint occurrence of precipitation and temperature extremes across India.

## Conclusions and outlook

This study reveals a notable spatio-temporal change in the compound extreme events based on precipitation and temperature across India. Significant increasing/decreasing trend of different (total six) compound extreme events (assessed through Joint Extreme Indices (JEIs)) are found to be more prominent after the climate regime shift in 1970s. Considering the spatial extent, areas exhibiting cold and wet spell have been substantially decreased in the country. The recent decades since the 2000s have witnessed stronger increase in the spatial extent of above-normal hot and wet events. It is further noticed that either precipitation or temperature extremes alone did not manifest such exceptional changes in the spatial extent as compared to their joint indices.

Hot nights and wet days (HNWD) have been significantly increased across the country during the post climate regime shift period. More increase is noticed in the joint extreme involving hot nights than that involving hot days. This indicates that the temperature rise may have caused night temperature to rise more than day time temperature. Warm and dry spell (WDS) is found to increase even before the climate shift, i.e. before 1975 too.

Using the CMIP6 climate model projections and the extrapolated historical trend (if it continues at the same rate that observed in the past), the probable future changes in the spatial coverage are assessed separately. The results indicate that occurrences of some of the compound extremes will be more frequent and the spatial extent exhibiting such changes will gradually spread over the entire country, whereas a few may not be experienced anywhere. For example, frequent occurrences of extreme hot and wet days are expected to cover the entire country and in contrast, cold and wet spell event will drop below-normal everywhere across India before the end of this century. Temperature rise and associated increase in water holding capacity of the atmosphere triggers the precipitation extremes. Besides, changes in land–atmosphere interactions and alterations in the large scale climate patterns owing to the gradually warming climate also contribute towards amplifying the precipitation and temperature extremes during the recent past. These are perhaps the most acceptable justification behind the change in compound extreme events as revealed in this study.

This study opens up multiple future studies. For instance, how does the large-scale coupled oceanic–atmospheric circulations affect the change in the characteristics of the compound extremes? Is there any such link or these are mostly influenced by the local factors, pertaining to climate change. Secondly, the well-known Clausius–Clapeyron scaling principle suggests that the precipitation increases with temperature rise as a result of increase in the water holding capacity of the atmosphere. However, this consensus may vary across different climatic and geographic locations under the influence of various factors. How these variables are related, and how exactly the changing pattern of one follows another needs to be explored across Indian mainland.

### Data and methodology

#### Observed data

Daily gridded precipitation data with spatial resolution of 0.25° (latitude) × 0.25° (longitude) and temperature (maximum and minimum) data with spatial resolution of 1° (latitude) × 1° (longitude) across Indian mainland are obtained from India Meteorological Department (IMD) (https://www.imdpune.gov.in/Clim_Pred_LRF_New/Grided_Data_Download.html, assessed in April, 2021) for the period 1951–2019. Precipitation data is upscaled to 1° (latitude) × 1° (longitude) spatial resolution to match with temperature data through inverse distance weighted interpolation (IDW) method.

The gridded daily rainfall data used in this study was developed by Pai et al.^53^. Towards this, the daily rainfall records from 6955 rain gauge stations with varying availability periods were used, which is the highest number of stations used by any studies so far for preparing the gridded rainfall data over India. Out of these 6955 stations, 547 were IMD observatory stations, 494 were hydro-meteorology observatories and 74 are agromet observatories^54,55^. The remaining are rainfall reporting stations maintained by the State Governments. On an average, data from about 2600 stations per year were available for the preparation of gridded data. However, the data density varied from year to year from about 1450 in the first year (1901) to about 3950 during the period 1991–1994. The density was relatively higher (≥ 3100 stations per day) from 1951 onwards^56^. In regard to region wise difference across the country, the density of the stations is relatively high in the south Peninsular and relatively low over northern most areas of the country, northwest India, northeast India, and eastern parts of central India. The station data were gridded by using the inverse distance weighted interpolation (IDW) method. Prior to the gridding, quality control measures such as check for missing data, duplicate station check, extreme value check etc. are applied on the station point rainfall data.

The gridded temperature dataset was developed using the daily minimum (night time) and maximum (day time) temperature data from 395 synoptic stations spread uniformly over the country^57,58^. The station data is interpolated to grids using the modified version of Shepard’s angular distance weighting algorithm^59^. In order to avoid biases in the gridding, daily temperature anomalies were used instead of absolute values. Towards this, climatological normal of maximum and minimum temperatures for the period 1971–2000 was calculated for each station. Prior to the interpolation, preliminary quality controls such as removing outliers and ensuring homogeneity were employed on the station data. Also, it was ensured that all the stations have the same data length to avoid errors due to inhomogeneity in station density.

#### Global climate model data from CMIP6

Multimodel projections from CMIP6 archive (https://esgfnode.llnl.gov/search/cmip6/) are used in this study. A total of eight Global Climate Models (GCMs) are selected and the daily precipitation, maximum temperature and minimum temperature data accessed. Only the first realization (r1i1p1) from each model is used. Details of the selected GCMs are given in Table S1, along with their respective modelling groups, countries/regions and horizontal resolution. These models have been commonly applied in hydroclimatology related studies^60–63^. Prior to the analysis, the data from each model were re-gridded from original spatial resolutions to a 1° × 1° common grid resolution in order to make it coincide with that of the observed temperature and precipitation data. To accomplish this, the Inverse Distance Weighting (IDW) method is applied.

Owing to differences in the feedback processes among different climate models, the response of models to future Greenhouse Gas (GHG) emissions, is subject to large uncertainties which affect the reliability of the estimate. Therefore the modelling of carbon emissions has been eliminated in CMIP6 and replaced by the Scenario Model Intercomparison Project (Scenario MIP)^64^. The scenarios are the updated combination of Shared Socioeconomic Pathways (SSPs)^60^ and forcing levels of the Representative Concentration Pathways (RCP), such as: SSP1-2.6 (+ 2.6 W m^−2^; low forcing sustainability pathway), SSP2-4.5 (+ 4.5 W m^−2^; medium forcing middle-of-the-road pathway), SSP3-7.0 (+ 7.0 W m^−2^; medium- to high-end forcing pathway), and SSP5-8.5 (+ 8.5 W m^−2^; high-end forcing pathway)^65^. Among these scenarios, the SSP5-8.5 can be considered as the worst scenario for the future which embodies the impact of unconventional socio-economic development^66^ and is the considered scenario for this study.

#### Precipitation and temperature extreme indices

A total of three Precipitation Extreme Indices (PEIs) and four Temperature Extreme Indices (TEIs) are considered. Most of them are listed in the Expert Team on Climate Change Detection, Monitoring and Indices (ETCCDI)^67,68^. It can be noted that the precipitation indices developed by ETCCDI expressing frequency of extreme precipitation are based on fixed value thresholds only. However, considering the huge variation in local precipitation climatology for a vast country like India, we added Extreme Wet Days (EWD) index, which is based on percentile threshold. Details of all the indices based on individual variable (precipitation or temperature) are shown in Tables S2 and S3 in the supplementary document.

#### Joint Extreme Indices

**Figure 8:**
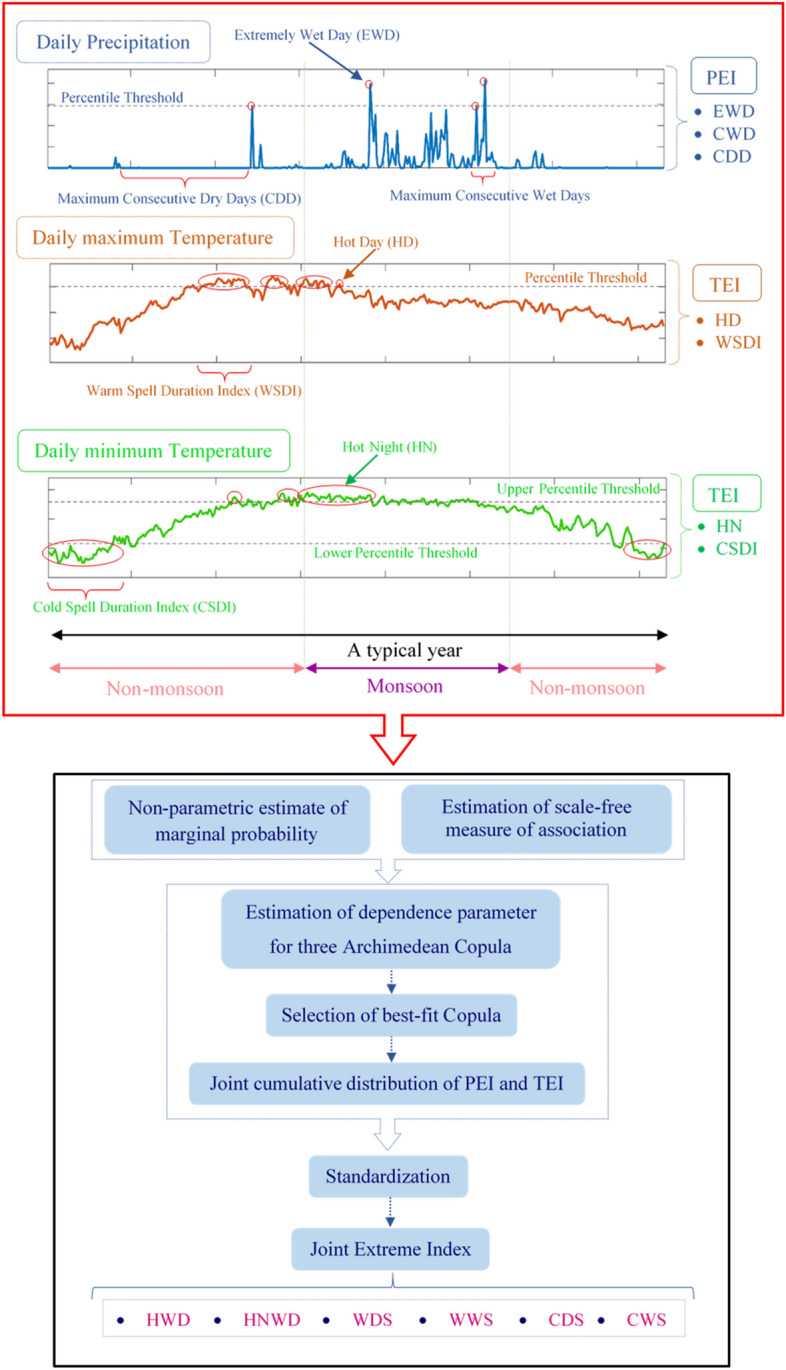
A schematic outline of methodology to develop the Joint Extreme Indices (JEIs).

A general methodological approach to develop the Joint Extreme Indices (JEIs) is shown in Fig. 8. Different combinations of the PEIs and TEIs are utilized to construct six JEIs, which are shown in Table 1. In order to develop joint distribution function of PEI and TEI, we utilized bivariate copulas. Copula provides a convenient way to deal with multivariate phenomena as it enables the construction of the joint distribution in a flexible way in which the marginal distributions are independent of the dependence structure modelling, existing between the considered random variables^41,69,70^. Previous studies have indicated that copulas perform well for bivariate problems in several fields including hydroclimatology^71–75^.

The detailed theoretical background and descriptions regarding the use of copulas can be found in Nelsen^40^ and Salvadori et al.^76^. A brief description on copula theory is as follows: a copula is a function that joins multiple distribution functions to their one-dimensional marginal distribution functions^77^. According to Sklar’s theorem, every joint distribution can be written in terms of a copula and its univariate marginal distributions^78^. For instance, two random variables $X$ (here, PEI) and $Y$ (here, TEI) with cumulative distribution functions ${F}_{X}\left(x\right)=P\left(X\le x\right)$ and ${F}_{Y}\left(y\right)=P\left(Y\le y\right)$, the joint cumulative distribution of $X$ and $Y$ can be expressed as,

1 $${F}_{X,Y}\left(x,y\right)=P\left(X\le x,Y\le y\right)=C\left({F}_{X}\left(x\right),{F}_{Y}\left(y\right); \theta \right)$$

where $C$ denotes the copula function of reduced variates or marginal distributions i.e. $u={F}_{X}\left(x\right)$ and $v={F}_{Y}\left(y\right)$ of the corresponding random variables $X$ and $Y$, such that for all *x, y* in $\overline{R }\in \left(-\infty ,\infty \right)$, and $\theta$ is parameter of the copula.

Nonparametric estimates of the marginal probability are obtained by using kernel density estimator^79^. The kernel estimate of for a real-valued time series, ${x}_{i}, i=1, 2, \dots n$ *,* can be expressed as,

2 $${\widehat{f}}_{X}\left(x\right)=\frac{1}{n}\sum_{i=1}^{n}{K}_{h}\left(x-{x}_{i}\right)$$

where $K_{h} \left( z \right) = \frac{1}{h}Kr\left( {\frac{z}{h}} \right)$, in which *h* is the smoothing parameter and *Kr* is the kernel function. Mathematical formulations of the kernel functions can be found in Bosq^80^. The association between the random variables is measured by Kendall rank correlation coefficient, which gives scale-free measure of association.

To model various kinds of dependence between the associated random variables, there exist different copula functions. In particular, several families of Archimedean copulas have been popular choices for dependence models because of their simplicity and versatility^4,76^. Here, three Archimedean copulas, namely, Frank, Clayton, and Gumbel-Hougaard, having an extensive use in hydroclimatic applications^4^, are adopted. Mathematical details of these copulas are provided briefly in Table S4 and the comprehensive description can be found in Nelsen^40^.

Out of the bivariate alternate copulas, the best-fit copula is selected based on goodness-of-fit test using two statistics, namely Kolmogorov–Smirnov (*T*_*n*_) and Cramér–von Mises (*S*_*n*_)^77^. These statistics are based on the distance between the fitted parametric copula (${C}_{n}^{\theta })$ and empirical copula $({C}_{n})$. The empirical copula function ${C}_{n}$ is defined by:

3 $${C}_{n}\left(u,v\right)=\frac{1}{n}\sum_{\forall u,v}\mathfrak{I}\left[\left(U\le u\right),\left(V\le v\right)\right], u,v \in I$$

where n is the count of data values and $\mathfrak{I}$ (·) is the indicator function that takes a value of 1 if the argument (•) is true and 0 if it is false. The statistics ${S}_{n}$ and ${T}_{n}$ are expressed as:

4 $${S}_{n}=\sum_{\forall u,v}{\left({C}_{n}\left(u,v\right)-{C}_{n}^{\theta }\left(u,v\right)\right)}^{2}$$

5 $${T}_{n}=max\left|\sqrt{n}\left({C}_{n}\left(u,v\right)-{C}_{n}^{\theta }\left(u,v\right)\right)\right|$$

Lower values of these statistics indicate a better fit. After obtaining the best fit copula, the joint probability distribution is constructed using Eq. (1).

To frame Joint Extreme Index (JEI), the joint probability is standardized based on normal quantile transformation (NQT) technique^81^. In this technique, the joint non-exceedance probability is transformed through the inverse standard normal distribution. Thus, the JEI is mathematically expressed as:

6 $$\mathrm{JEI}={\Phi }^{-1}\left({F}_{X,Y}\left(x,y\right)\right)$$

where ${\Phi }^{-1}$ denotes the inverse function of the standard normal distribution.

Here, it can be noted that the risk associated with the compound climate extremes depends on mutual interaction among multiple variables and the occurrence of their extremes resulting in amplified impacts^44^. Towards this, copulas with higher dimensions may be opted, if necessary^82^. Thus, further modelling and analysis of compound extremes from different combinations of crucial hydroclimate variables considering the changing climate scenarios is kept as a future scope of the study.

#### Annual and seasonal variation

The joint extreme indices are developed considering three different cases with respect to data from a particular year: (1) using data from entire year, (2) using data from monsoon (June through September) season only and (3) using data from non-monsoon (October through May) season only. The annual along with the season-wise evaluation of concurrent precipitation and temperature extreme characteristics is helpful owing to the substantial seasonal variation in precipitation and temperature across India. During the monsoon season, major portion of the annual precipitation is received across many parts of India, however, with great spatial variation. During the non-monsoon season various regional conditions and weather systems involving different hydrometeorological variables including temperature, play vital role towards the evolution and execution of monsoon precipitation^83^. Further, significant changes in extreme precipitation characteristics are reported beyond the monsoon season, across most of the country^84,85^. Similarly, the temperature extremes, across different regions of the county are changing significantly during the monsoon as well as non-monsoon seasons in the recent years^86,87^.

## Supplementary Information

Supplementary Information 1.
