# Supplementary material for: Revealing alarming changes in spatial coverage of joint hot and wet extremes across India
Source: Sci Rep. 2021 Sep 9;11:18031. doi: 10.1038/s41598-021-97601-z (PMC8429548; doi:10.1038/s41598-021-97601-z)
Supplement: Supplementary file 1 — Supplementary Information 1. [file 41598_2021_97601_MOESM1_ESM.docx]

Supplementary Document

**Revealing alarming changes in spatial coverage of joint hot and wet extremes across India**

Subhasmita Dash and Rajib Maity

Department of Civil Engineering, Indian Institute of Technology Kharagpur, Kharagpur – 721302, West Bengal, India

Corresponding author: Dr. Rajib Maity, email IDs: [rajib@civil.iitkgp.ac.in](mailto:rajib@civil.iitkgp.ac.in), [rajibmaity@gmail.com](mailto:rajibmaity@gmail.com).

This document contains additional figures and tables as mentioned in the main article. The figures provide details on the additional analysis carried out to reach to the conclusions. List of figures and tables in order of their presentation are as follows:

| Figure S1: | Map of the four homogeneous rainfall zones in India, namely North West India, North East India, Central India and South Peninsular India, as defined by India Meteorological Department projected onto 1° × 1° daily rainfall grids. |
| --- | --- |
| Figure S2: | Relationship between Joint Extreme Index (JEI) and its constituting indices, i.e. Precipitation Extreme Index (PEI) and Temperature Extreme Index (TEI). (a) Time series of PEI (here, CDD), TEI (here, WSDI) and their corresponding JEI (here, WDS) over the period 1975-2019 for South Peninsular India (SPI), (b) same as (a) but for North West India (NWI), (c) Scatter plot of JEI on the phase plane of its constituting precipitation and temperature extreme indices. Size and color of the circle markers indicate magnitude and phase of JEI, respectively for South Peninsular India (SPI), and (d) same as (c) but for North West India (NWI). |
| Figure S3: | Relationship between Joint Extreme Index (JEI) and its constituting indices, i.e. Precipitation Extreme Index (PEI) and Temperature Extreme Index (TEI). (a) Time series of PEI (here, CWD), TEI (here, WSDI) and their corresponding JEI (here, WWS) over the period 1975-2019 for South Peninsular India (SPI), (b) same as (a) but for North West India (NWI), (c) Scatter plot of JEI on the phase plane of its constituting precipitation and temperature extreme indices. Size and colour of the circle markers indicate magnitude and phase of JEI, respectively for South Peninsular India (SPI), and (d) same as (c) but for North West India (NWI). |
| Figure S4: | Relationship between Joint Extreme Index (JEI) and its constituting indices, i.e. Precipitation Extreme Index (PEI) and Temperature Extreme Index (TEI). (a) Time series of PEI (here, EWD), TEI (here, TN90) and their corresponding JEI (here, HNWD) over the period 1975-2019 for South Peninsular India (SPI), (b) same as (a) but for North West India (NWI), (c) Scatter plot of JEI on the phase plane of its constituting precipitation and temperature extreme indices. Size and colour of the circle markers indicate magnitude and phase of JEI, respectively for South Peninsular India (SPI), and (d) same as (c) but for North West India (NWI). |
| Figure S5: | Relationship between Joint Extreme Index (JEI) and its constituting indices, i.e. Precipitation Extreme Index (PEI) and Temperature Extreme Index (TEI). (a) Time series of PEI (here, CDD), TEI (here, CSDI) and their corresponding JEI (here, CDS) over the period 1975-2019 for South Peninsular India (SPI), (b) same as (a) but for North West India (NWI), (c) Scatter plot of JEI on the phase plane of its constituting precipitation and temperature extreme indices. Size and colour of the circle markers indicate magnitude and phase of JEI, respectively for South Peninsular India (SPI), and (d) same as (c) but for North West India (NWI). |
| Figure S6: | Relationship between Joint Extreme Index (JEI) and its constituting indices, i.e. Precipitation Extreme Index (PEI) and Temperature Extreme Index (TEI). (a) Time series of PEI (here, CWD), TEI (here, CSDI) and their corresponding JEI (here, CWS) over the period 1975-2019 for South Peninsular India (SPI), (b) same as (a) but for North West India (NWI), (c) Scatter plot of JEI on the phase plane of its constituting precipitation and temperature extreme indices. Size and colour of the circle markers indicate magnitude and phase of JEI, respectively for South Peninsular India (SPI), and (d) same as (c) but for North West India (NWI). |
| Figure S7: | Future projection of observed significant change (during 1975-2019) in spatial extent (in proportion of entire Indian mainland) exhibiting positive Joint Extreme Indices (JEIs): a) Warm and Wet Spell (WWS) and b) Warm and Dry Spell (WDS) considering entire year. |
| Figure S8: | Future projection of observed significant change (during 1975-2019) in spatial extent (in proportion of entire Indian mainland) exhibiting positive Joint Extreme Indices (JEIs): a) Cold and Wet Spell (CWS), b) Warm and Wet Spell (WWS), c) Hot Nights and Wet Days (HNWD), considering the monsoon season. |
| Figure S9: | Future projection of observed significant change (during 1975-2019) in spatial extent (in proportion of entire Indian mainland) exhibiting positive Joint Extreme Indices (JEIs): a) Cold and Wet Spell (CWS), b) Warm and Dry Spell (WDS), c) Hot Nights and Wet Days (HNWD), and d) Hot and Wet Days (HWD), considering the non-monsoon season. |
| Figure S10: | Spatial distribution of a) Warm and Wet Spell (WWS) and b) Warm and Dry Spell (WDS) across India during the future period i.e., 2075-2084. |
| Figure S11: | Spatial distribution of a) Cold and Wet Spell (CWS), b) Warm and Wet Spell (WWS) and c) Hot Nights and Wet Days (HNWD) considering the monsoon season across India during the future period i.e., 2075-2084. |
| Figure S12: | Spatial distribution of a) Cold and Wet Spell (CWS), b) Warm and Dry Spell (WDS), c) Hot Nights and Wet Days (HNWD), and d) Hot and Wet Days (HWD), considering the non-monsoon season across India during the future period i.e., 2075-2084. |
| Figure S13: | Year-wise variation of areal coverage (in proportion of entire Indian mainland) exhibiting positive Precipitation and Temperature Extreme Indices (PEIs and TEIs) during the post-climate regime shift period, i.e. 1975-2019 for three cases considering entire year (shown in solid shaded lines), monsoon season (shown in black lines with circle markers) and non-monsoon season (shown in black lines with star markers): (a) CWD, (b) CDD, (c) EWD, (d) CSDI, (e) WSDI, (f) TN90 and (g) TX90. Rate of changes are shown in the legend of the respective index and the significant rate of change (as per M-K test at 5% significance) are noted with star markers. |

| Table S1: | Global Climate Models from CMIP6 archive along with corresponding Institutions, Country of origin and horizontal resolution. |
| --- | --- |
| Table S2: | Details of Precipitation Extreme Indices (PEIs) |
| Table S3: | Details of Temperature Extreme Indices (TEIs) |
| Table S4: | Details of three considered Archimedean copulas |

**Figure S1: Map of the four Indian (mainland India) homogeneous rainfall zones, namely North West India, North East India, Central India and South Peninsular India, as defined by India Meteorological Department projected onto 1° × 1° daily rainfall grids.**

**Figure S2: Relationship between Joint Extreme Index (JEI) and its constituting indices, i.e. Precipitation Extreme Index (PEI) and Temperature Extreme Index (TEI). (a) Time series of PEI (here, CDD), TEI (here, WSDI) and their corresponding JEI (here, WDS) over the period 1975-2019 for South Peninsular India (SPI), (b) same as (a) but for North West India (NWI), (c) Scatter plot of JEI on the phase plane of its constituting precipitation and temperature extreme indices. Size and colour of the circle markers indicate magnitude and phase of JEI, respectively for South Peninsular India (SPI), and (d) same as (c) but for North West India (NWI).**

**Figure S3: Relationship between Joint Extreme Index (JEI) and its constituting indices, i.e. Precipitation Extreme Index (PEI) and Temperature Extreme Index (TEI). (a) Time series of PEI (here, CWD), TEI (here, WSDI) and their corresponding JEI (here, WWS) over the period 1975-2019 for South Peninsular India (SPI), (b) same as (a) but for North West India (NWI), (c) Scatter plot of JEI on the phase plane of its constituting precipitation and temperature extreme indices. Size and colour of the circle markers indicate magnitude and phase of JEI, respectively for South Peninsular India (SPI), and (d) same as (c) but for North West India (NWI).**

**Figure S4: Relationship between Joint Extreme Index (JEI) and its constituting indices, i.e. Precipitation Extreme Index (PEI) and Temperature Extreme Index (TEI). (a) Time series of PEI (here, EWD), TEI (here, TN90) and their corresponding JEI (here, HNWD) over the period 1975-2019 for South Peninsular India (SPI), (b) same as (a) but for North West India (NWI), (c) Scatter plot of JEI on the phase plane of its constituting precipitation and temperature extreme indices. Size and colour of the circle markers indicate magnitude and phase of JEI, respectively for South Peninsular India (SPI), and (d) same as (c) but for North West India (NWI).**

**Figure S5: Relationship between Joint Extreme Index (JEI) and its constituting indices, i.e. Precipitation Extreme Index (PEI) and Temperature Extreme Index (TEI). (a) Time series of PEI (here, CDD), TEI (here, CSDI) and their corresponding JEI (here, CDS) over the period 1975-2019 for South Peninsular India (SPI), (b) same as (a) but for North West India (NWI), (c) Scatter plot of JEI on the phase plane of its constituting precipitation and temperature extreme indices. Size and colour of the circle markers indicate magnitude and phase of JEI, respectively for South Peninsular India (SPI), and (d) same as (c) but for North West India (NWI).**

**Figure S6: Relationship between Joint Extreme Index (JEI) and its constituting indices, i.e. Precipitation Extreme Index (PEI) and Temperature Extreme Index (TEI). (a) Time series of PEI (here, CWD), TEI (here, CSDI) and their corresponding JEI (here, CWS) over the period 1975-2019 for South Peninsular India (SPI), (b) same as (a) but for North West India (NWI), (c) Scatter plot of JEI on the phase plane of its constituting precipitation and temperature extreme indices. Size and colour of the circle markers indicate magnitude and phase of JEI, respectively for South Peninsular India (SPI), and (d) same as (c) but for North West India (NWI).**

**Figure S7: Future projection of observed significant change (during 1975-2019) in spatial extent (in proportion of entire Indian mainland) exhibiting positive Joint Extreme Indices (JEIs): a) Warm and Wet Spell (WWS) and b) Warm and Dry Spell (WDS) considering entire year.**

**Figure S8: Future projection of observed significant change (during 1975-2019) in spatial extent (in proportion of entire Indian mainland) exhibiting positive Joint Extreme Indices (JEIs): a) Cold and Wet Spell (CWS), b) Warm and Wet Spell (WWS), c) Hot Nights and Wet Days (HNWD), considering the monsoon season.**

**Figure S9: Future projection of observed significant change (during 1975-2019) in spatial extent (in proportion of entire Indian mainland) exhibiting positive Joint Extreme Indices (JEIs): a) Cold and Wet Spell (CWS), b) Warm and Dry Spell (WDS), c) Hot Nights and Wet Days (HNWD), and d) Hot and Wet Days (HWD), considering the non-monsoon season.**

**Figure S10: Spatial distribution of a) Warm and Wet Spell (WWS) and b) Warm and Dry Spell (WDS) across India during the future period i.e., 2075-2084. The figure was produced using MATLAB software (version R2021a, URL:** [**https://in.mathworks.com**](https://in.mathworks.com)**).**

**Figure S11: Spatial distribution of a) Cold and Wet Spell (CWS), b) Warm and Wet Spell (WWS) and c) Hot Nights and Wet Days (HNWD) considering the monsoon season across India during the future period i.e., 2075-2084. The figure was produced using MATLAB software (version R2021a, URL:** [**https://in.mathworks.com**](https://in.mathworks.com)**).**

**Figure S12: Spatial distribution of a) Cold and Wet Spell (CWS), b) Warm and Dry Spell (WDS), c) Hot Nights and Wet Days (HNWD), and d) Hot and Wet Days (HWD), considering the non-monsoon season across India during the future period i.e., 2075-2084. The figure was produced using MATLAB software (version R2021a, URL:** [**https://in.mathworks.com**](https://in.mathworks.com)**).**


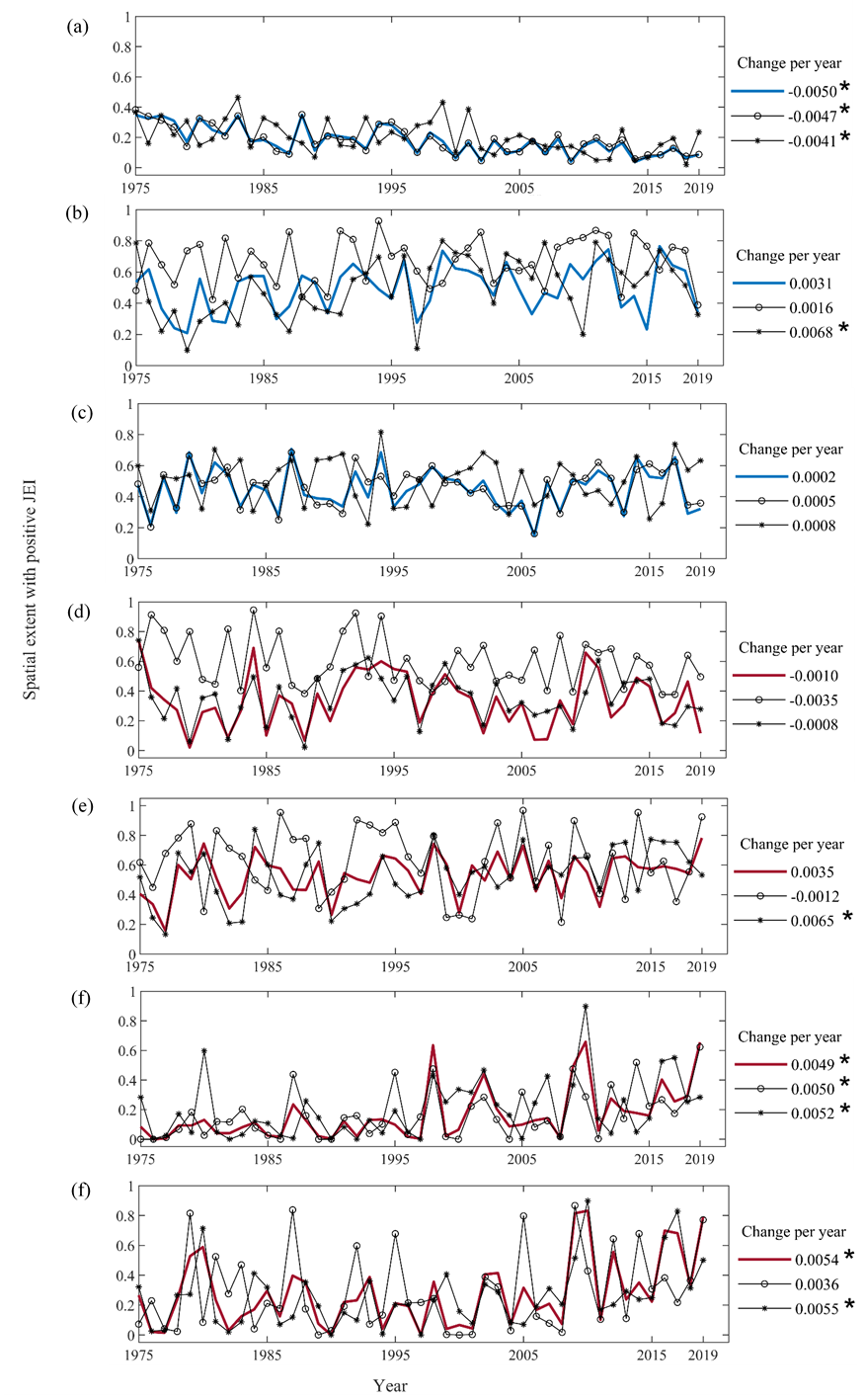


**Figure S13: Year-wise variation of areal coverage (in proportion of entire Indian mainland) exhibiting positive Precipitation and Temperature Extreme Indices (PEIs and TEIs) during the post-climate regime shift period, i.e. 1975-2019 for three cases considering entire year (shown in solid shaded lines), monsoon season (shown in black lines with circle markers) and non-monsoon season (shown in black lines with star markers): (a) CWD, (b) CDD, (c) EWD, (d) CSDI, (e) WSDI, (f) TN90 and (g) TX90. Rate of changes are shown in the legend of the respective index and the significant rate of change (as per M-K test at 5% significance) are noted with star markers.**

**Table S1:** **Global Climate Models from CMIP6 archive along with corresponding Institutions, Country of origin and horizontal resolution.**

| No. | Model | Modelling group (Country) | Resolution |
| --- | --- | --- | --- |
| 1 | AWI-CM-1-1-MR | Alfred Wegener Institute (Germany) | 0.9° ×0.9° |
| 2 | BCC-CSM2-MR | Beijing Climate Center (China) | 1.1° ×1.1° |
| 3 | CanESM5 | Canadian Centre for Climate Modelling and Analysis (Canada) | 2.8° ×2.8° |
| 4 | EC-Earth3-Veg | EC-Earth-Consortium (Europe) | 0.7° ×0.7° |
| 5 | EC-Earth3 |  |  |
| 6 | INM-CM4-8 | Institute for Numerical Mathematics (Russia) | 2° ×1.5° |
| 7 | INM-CM5-0 |  |  |
| 8 | MPI-ESM1-2-HR | Max-Planck-Institute for Meteorology (Germany) | 0.9° ×0.9° |

**Table S2: Details of Precipitation Extreme Indices (PEIs)**

| Index Id | Index name | Description |
| --- | --- | --- |
| EWD | Extreme Wet Days | Days with precipitation >= 90^th^ percentile of the daily precipitation amount during the base period, 1980-2010 |
| CDD | Consecutive Dry Days | Maximum number of consecutive days with precipitation <1.00 mm |
| CWD | Consecutive Wet Days | Maximum number of consecutive days with precipitation ≥ 1.00 mm |

**Table S3: Details of Temperature Extreme Indices (TEIs)**

| Index Id | Index name | Description |
| --- | --- | --- |
| TX90 | Hot Days | Number days when daily maximum temperature, TX > 90^th^ percentile (corresponding to the TX during the base period 1980-2010) |
| TN90 | Hot Nights | Number days when daily maximum temperature, TN > 90^th^ percentile (corresponding to the TN during the base period 1980-2010) |
| WSDI | Warm Spell Duration Index | Number of days with at least 6 consecutive days when daily maximum temperature, TX > 90^th^ percentile (corresponding to the TX during the base period 1980-2010) |
| CSDI | Cold Spell Duration Index | Number of days with at least 6 consecutive days when daily minimum temperature, TN < 10^th^ percentile (corresponding to the TN during the base period 1980-2010) |

**Table S4: Details of three considered Archimedean copulas**

| Copula Type | Copula Function, $C(u,v)$ | Copula Parameter, $\theta$ |
| --- | --- | --- |
| Frank | $\frac{-1}{\theta}\ln\left(1+\frac{\left(e^{-\theta u}-1\right)\left(e^{-\theta v}-1\right)}{\left(e^{-\theta}-1\right)}\right)$ | $\left(-\infty,\infty\right)\backslash\left\{0\right\}$ |
| Clayton | $\left[\max\left(u^{-\theta}+v^{-\theta}-1,0\right)\right]^{\frac{-1}{\theta}}$ | $\left[-1,\infty\right)\backslash\left\{0\right\}$ |
| Gumbel | $\exp\left(-\left[\left(-\ln u\right)^{\theta}+\left(-\ln v\right)^{\theta}\right]^{\frac{1}{\theta}}\right)$ | $\left[1,\infty\right)$ |
